# Relaxation Effect of Patchouli Alcohol in Rat Corpus Cavernous and Its Underlying Mechanisms

**DOI:** 10.1155/2020/3109069

**Published:** 2020-02-28

**Authors:** Fangjun Chen, Yifei Xu, Jing Wang, Xufeng Yang, Hongying Cao, Ping Huang

**Affiliations:** ^1^School of Pharmaceutical Sciences, Guangzhou University of Chinese Medicine, Guangzhou, Guangdong 510006, China; ^2^Shenzhen Traditional Chinese Medicine Hospital, The Fourth Clinical Medical College of Guangzhou University of Chinese Medicine, Shenzhen 518033, China; ^3^Dongguan & Guangzhou University of Chinese Medicine Cooperative Academy of Mathematical Engineering for Chinese Medicine, Guangzhou University of Chinese Medicine, Guangzhou, Guangdong 510006, China

## Abstract

In this study, we investigated the relaxation effect and mechanisms of patchouli alcohol (PA) on rat corpus cavernosum. Corpus cavernosum strips were used in organ baths for isometric tension studies. The results showed that PA demonstrated concentration-dependent relaxation effect on rat corpus cavernosum. The relaxant response to PA was not influenced by tetrodotoxin and atropine while it was significantly inhibited by removal of endothelium. L-N^G^-nitroarginine methyl ester (L-NAME, a nitric oxide synthase inhibitor) or 1H-[1,2,4]oxadiazolo[4,3-a]quinoxalin-1-one (ODQ, a soluble guanylate cyclase inhibitor) significantly inhibited relaxation response to PA, whereas indomethacin (COX inhibitor) had no effect on PA-induced relaxation. The treatment of endothelium-deprived corpus cavernosum with several potassium channel blockers including tetraethylammonium (TEA), 4-aminopyridine (4-AP), and glibenclamide had no effect on PA-induced relaxation. Endothelium-deprived corpus cavernosal contractions induced by cumulative addition of Ca^2+^ to high KCl solution without CaCl_2_ were significantly inhibited by PA. Also, PA improved relaxant capacity of sildenafil in rat corpus cavernosum. In addition, the perfusion with PA significantly increased the levels of cGMP and expression of mRNA and protein of neuronal nitric oxide synthase (nNOS) and endothelial nitric oxide synthase (eNOS). Furthermore, intracavernous injection of PA enhanced the rise in intracavernous pressure in rats during cavernosal nerve electric stimulation. In conclusion, PA relaxed the rat corpus cavernosum attributed to both endothelium-dependent and -independent properties. While the former component was mostly involved in nitric oxide signaling pathway, the endothelium-independent mechanism involved in PA-induced relaxation was probably linked to calcium antagonism.

## 1. Introduction

Erectile dysfunction is the inability of the penis to achieve and maintain enough erection to obtain a satisfactory sex life for a period of at least 6 months [1]. Erectile dysfunction has presently emerged as the most important disease affecting men worldwide. Approximately 322 million men worldwide have been predicted to suffer from erectile dysfunction by 2025 [2].

Regular penile erection is a neuromodulated vascular response. Stimulation of neural signal is transmitted from the hypothalamic erectile center to the cavernous nerve and conducted to the penile tissue through nonadrenal noncholinergic nerve, which promotes the release of nitric oxide (NO) from nerve endings and endothelial cells. NO activates guanylyl cyclase resulting in an increase of cyclic guanosine monophosphate (cGMP) production. Then, corpus cavernosum smooth muscle relaxes and penis erects [3]. Erectile dysfunction is often a disease of vascular origin caused by impairment of endothelium-dependent or endothelium-independent smooth muscle relaxation [4]. Phosphodiesterase-5 (PDE-5) inhibitors are used extensively to treat erectile dysfunction [5]. However, the effects of PDE-5 inhibitors were proved to rely on the integrity of the NO production system. Thus, these drugs are less efficient in diabetic patients [6]. Patients treated with nitrate, nitrate donors, or vasodilators should not take PDE-5 inhibitors together for the potential hypotension hazard [6, 7]. In addition, these drugs are costly, and some side effects such as headache and giddiness are always associated with the use of PDE-5 inhibitors [8], which suggests that new pharmacological methods should be explored in clinical treatment.

**Figure 1:**
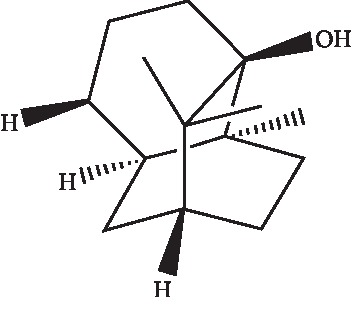
Structure of patchouli alcohol.

Pogostemonis Herba, the dry aerial part of *Pogostemon cablin* (Blanco) Benth., is a well-known traditional medicine herb used to treat functional gastrointestinal diseases in many Asian countries [9]. Patchouli alcohol (PA; its structure is shown in Figure 1) is a tricyclic sesquiterpene and one of the major bioactive ingredients. This compound is mainly isolated from Pogostemon Herba. In European countries, PA is widely used in daily products and perfume industries [10]. Modern studies have revealed that Pogostemonis Herba and PA exhibited anti-inflammatory, immunomodulation, antibacterial, and antioxidant activities [11–15]. Recent research indicated that PA can improve diarrhea-predominant irritable bowel syndrome and has antidepressant effect through the NO signaling pathway [16, 17]. Our previous study showed that PA can increase NO release in rat corpus cavernosum smooth muscle by using DAF staining method (data not shown). In this follow-up study, we attempted to further illustrate the underlying mechanism of PA relaxation of the corpus cavernosum.

## 2. Materials and Methods

### 2.1. Animals

This study was registered at the Animal Care and Use Committee of the Guangzhou University of Chinese Medicine. 12-week male Sprague-Dawley rats (250–350 g; Medical Experimental Animal Center of Guangzhou University of Chinese Medicine, China) were housed under standard animal laboratory conditions (12 h/12 h light/dark cycle, at 22 ± 1°C and under relative humidity 62 ± 5%).

### 2.2. Drugs and Solutions

Phenylephrine, carbachol, tetrodotoxin, atropine, indomethacin, L-N^G^-nitroarginine methyl ester (L-NAME), 1H-[1,2,4]oxadiazolo[4,3-a]quinoxalin-1-one (ODQ), tetraethylammonium (TEA), 4-aminopyridine (4-AP), glibenclamide, and sildenafil were purchased from Target Molecule Company. CHAPS, CaCl_2_, KCl, and ethyleneglycol-bis-(b-amino-ethyl ether)-N,N,N′,N′-tetra-acetic acid (EGTA) were obtained from Sigma Chemical Company. Phenylephrine was dissolved in normal saline containing 0.1% ascorbic acid, ODQ and glibenclamide were dissolved in dimethyl sulphoxide (DMSO), and indomethacin was dissolved in ethanol. Stock solutions were stored at −20°C, and dilutions were prepared during the experiment.

PA (purity > 99%) was obtained as previously described and further confirmed by melting point, IR, ^1^H NMR and ^13^C NMR, and MS in our previous study [18]. In the organ bath experiments, PA dissolved in dimethyl sulphoxide (DMSO), and equal volume of vehicle was used as a control (DMSO < 0.2%). In experiment of intracavernous pressure measurement, PA dissolved in medium chain triglycerides, and equal volume of vehicle was used as a control. Krebs solution was composed of NaCl 120 mM, KCl 5.9 mM, NaHCO_3_ 25 mM, NaH_2_PO_4_ 1.2 mM, MgCl_2_ 1.2 mM, CaCl_2_ 2.5 mM, and dextrose 11.5 mM. In a calcium-free high KCl solution, CaCl_2_ was replaced with MgCl_2_, and 0.5 mM EGTA was added.

### 2.3. Tissue Preparation

After euthanasia by CO_2_ asphyxiation, the rat penis was excised along the pubis and placed in a Petri dish containing ice-cold (4°C) Krebs solution bubbled with a mixture of 95% O_2_ and 5% CO_2_. The penis was separated from the midshaft to the base of the crura. After the fascia tunica albuginea and corpus spongiosum were removed, the two corpus cavernosa strips were separated by cutting the fibrous septum between them. Each end of the corpus cavernosum smooth muscle strip was tied with silk, transferred to a 5-ml organ bath containing Krebs solution maintained at 37°C, and continuously gassed with carbogen (95% O_2_ + 5% CO_2_). One end of each strip was attached to a fixed hook, and the other end was connected to a force transducer (Harvard Apparatus Co. Ltd, USA) to measure isometric tension. The corpus cavernosum smooth muscle strips were stretched under 500 mg of tension and equilibrated for 60 min. Force measurements were displayed on PowerLab (AD Instrument Co. Ltd, Australia) and digitally acquired by computer (LabChart software). To obtain endothelium-deprived corpus cavernosum, corpus cavernosum endothelium was removed using detergent 0.3% CHAPS. For the endothelial integrity test, the corpus cavernosum strips treated with carbachol (1 *μ*M) that did not relax or relax poorly (<10% of the maximal relaxation) were considered to have endothelium removed.

For the *ex vivo* penile perfusion model [19], penis was rapidly excised from the pubic bone. Then, the urethra was dissected free from the penile. And the penises were frozen in liquid nitrogen after perfused with PA (20, 40, or 80 *μ*M) for 2 h in Krebs solution maintained at 37°C, and continuously gassed with carbogen. Afterwards, the tissues were used to measure the level of cGMP and cAMP. Moreover, quantitative real-time PCR and western blot assays were carried out to evaluate the gene and protein expressions of endothelial nitric oxide synthase (eNOS) and neuronal nitric oxide synthase (nNOS).

### 2.4. Organ Bath Experiments

After the equilibration for 60 min, the tissues were contracted with phenylephrine (10 *μ*M) and subjected to cumulative additions of PA (1, 10, 30, 100 *μ*M). These PA concentrations were selected based on pilot experiments. Tissues treated with the same volume of DMSO served as the control.

To investigate whether PA-induced relaxation on the cavernosal strip was caused by neuronal effect or involved in muscarine, the effects of PA on phenylephrine-evoked contraction in corpus cavernosum strips after preincubation with tetrodotoxin (1 *μ*M) and atropine (30 *μ*M) for 20 min were observed. The role of the endothelium in PA-induced relaxation was examined by comparing the magnitude of relaxation between the endothelium-intact and endothelium-deprived corpus cavernosum. Then, response to PA was examined in the absence or presence of NOS inhibitors L-NAME (100 *μ*M), soluble guanylate cyclase inhibitor ODQ (10 *μ*M), or cyclooxygenase inhibitor indomethacin (30 *μ*M) added 30 min before precontraction with phenylephrine.

In the following experiments, we had evaluated the endothelium-independent relaxation response in endothelium-deprived corpus cavernosum. First, the role of K^+^ permeability in the effect of PA was studied in high K^+^ medium (60 mM). To investigate the possible contribution of K^+^ channels to PA-induced corpus cavernosum relaxation, the endothelium-deprived corpus cavernosum response to PA was also examined in the absence or presence of Ca^2+^-activated K^+^ (K_Ca_) channels blocker TEA (10 *μ*M), voltage-dependent K^+^ (KV) channels blocker 4-AP (300 *μ*M), or ATP-sensitive K^+^ (KATP) channels blocker glibenclamide (10 *μ*M) added 30 min before the contraction with phenylephrine. To evaluate the relationships between PA-induced relaxation of corpus cavernosum and the block of Ca^2+^ channels, in a calcium-free high KCl solution, the endothelium-deprived corpus cavernosum strips were preincubated with PA (100 *μ*M) or vehicle control for 30 min, and then the concentration-response curves were constructed by accumulatively adding CaCl_2_ (10^−4^–10^−2.5^ mol·L^−1^). In addition, phenylephrine (10 *μ*M) was used to stimulate IP3-stimulated Ca^2+^ release from the sarcoplasmic reticulum in the absence of extracellular calcium (Ca^2+^-free Krebs solution). The tissues were equilibrated for 45 min and then the bathing solution was replaced with Ca^2+^-free Krebs solution. Then, corpus cavernosum strips' contraction response to phenylephrine was examined in the absence and presence of PA (100 *μ*M) added 30 min before contraction with phenylephrine. Amplitude of the phenylephrine- or Ca^2+^-induced contraction was expressed in mg per mg of tissue (mg/mg tissue).

To investigate the possible synergistic interaction between PA and sildenafil, rat corpus cavernosum relaxation responses to sildenafil (0.01–10 *μ*M) were observed in absence or presence of PA (20 *μ*M) added 30 min before precontraction with phenylephrine.

### 2.5. Measurement of cGMP and cAMP

The tissues were homogenized in an ice-cold RIPA buffer, and then the tissue lysates were centrifuged at 12000×g and 4°C for 15 min. The supernatants were collected, and cGMP and cAMP concentrations of the supernatant were measured using ELISA kits (Tianjin Anoric Biotechnology Co. Ltd, Tianjin, China) according to the manufacturer's instruction. And the protein concentrations were measured using BCA assay.

### 2.6. Detection of nNOS and eNOS mRNA Expression by RT-PCR

The total RNA of the corpus cavernosum from *ex vivo* penile perfusion model was extracted using TRIzol Reagent (Thermo Fisher Scientific, USA). RNA was reverse-transcribed using the cDNA synthesis kit (TIANGEN Biotech Co. Ltd, Beijing, China). The primer sequences were as follows: nNOS, 5′-GACCGAAGCTGGAAGAGGAACAAG-3′ (forward) and 5′-GTGTGGAGACGCACGAAGATGG-3′ (reverse); eNOS, 5′-CACAGGCATCACCAGGAAGAAGAC-3′ (forward) and 5′-TTCACACGCTTCGCCATCACC-3′ (reverse); actin, 5′-TCAGGTCATCACTATCGGCAAT-3′ (forward) and 5′-AAAGAAAGGGTGTAAAACGCA-3′ (reverse). PCR was performed on a CFX96 Touch™ Real-Time PCR Detection System (Bio-Rad Co. Ltd, USA) using Talent qPCR Pre-Mix (SYBR Green) kit (TIANGEN Biotech Co. Ltd, Beijing, China) with thermal cycles of 3 min at 95°C, 40 cycles of 5 s at 95°C, at 60°C for 15 s.

### 2.7. Western Blot Analysis of nNOS and eNOS Expression

The tissues were homogenized in an ice-cold RIPA buffer, and then the tissue lysates were centrifuged at 12000×g and 4°C for 15 min. The supernatants were collected, and the total protein concentrations were measured using BCA assay. An aliquot of 30 *µ*g of each protein sample was separated by electrophoresis on an 8% SDS-polyacrylamide gel and transferred onto a nitrocellulose membrane using a wet transfer system (LIUYI Biotech Co. Ltd, China). Nonspecific binding sites were blocked by 5% nonfat dry milk in 0.1% Tween-20 phosphate-buffered saline and then incubated overnight at 4°C with protein primary antibodies against eNOS (1 : 500; BD Transduction Laboratories Co. Ltd, USA), nNOS (1 : 500, BD Transduction Laboratories Co. Ltd, USA), and beta-actin (1 : 1000; 4A Biotech Co. Ltd, China). After washing, the membranes were incubated in horseradish peroxidase-conjugated goat antirabbit IgG (4A Biotech Co. Ltd, China) at room temperature for 2 h. The reaction antigen was detected with an enhanced chemiluminescence detection system (Millipore, USA) and visualized on Tanon-5200 Chemiluminescent Imaging System (Tanon Science & Technology Co. Ltd, China). Bands were quantitated by densitometry using ImageJ analyzer software.

### 2.8. Measurement of Intracavernous Pressure (ICP) in Rats

The 24 male Sprague-Dawley rats weighing 250–300 g were randomly divided into four groups (six rats per group): the control group (intracavernous injection of medium chain triglycerides) and three PA groups (intracavernous injection: 0.1, 0.2, and 0.4 mg/kg). The rats were anesthetized by intraperitoneal injection of pentobarbital sodium (40 mg/kg), and one carotid side was cannulated with an epidural anesthesia catheter connected to a three-way stopcock and used for the continuous monitoring of the arterial pressure and mean arterial pressure (MAP) via a pressure transducer (SP844; MEMSCAP Co. Ltd, France). The pressure measurements were displayed on PowerLab (AD Instrument Co. Ltd, Australia) and digitally acquired by a computer (LabChart software). The major pelvic ganglion was located at the posterolateral side of the prostate. The penile tunica albuginea was exposed after blunt dissection of the ischiocavernosus muscle. A blood collection needle connected to a three-way stopcock and filled with heparinized saline (250 U/ml) was introduced into the tunica albuginea for ICP recording. The cavernous nerve was stimulated using an electric stimulator and platinum electrodes after approximately 10 min of intracavernous injection of PA. The electrical stimulation parameters were set at 2.5 V, 12 Hz, and duration of 60 s. To examine the hemodynamic reaction in response to the autonomic nerve stimulation, the pressure was measured before, during, and after stimulation.

### 2.9. Data Analysis

Data are expressed as mean ± SEM. Responses to PA are expressed as percentages of the reversal of the tension developed in response to phenylephrine. The differences of means for the contractile or relaxation responses in the organ bath experiments were analyzed by *t*-test. In *in vivo* experiments, data were analyzed by one-way ANOVA. *P* < 0.05 was considered statistically significant.

## 3. Results

### 3.1. Responses to PA

**Figure 2:**
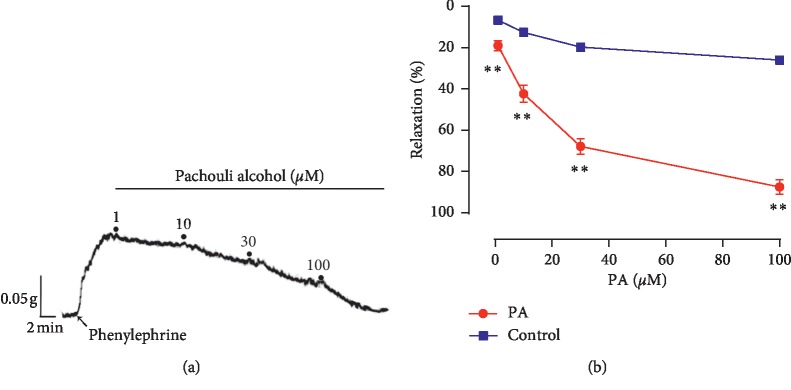
(a) Representative tracing showing relaxation of corpus cavernosum induced by PA (1–100 *μ*M) in rat corpus cavernosum after precontraction with phenylephrine. (b) Relaxation effects of PA on endothelium-intact corpus cavernosum precontracted with phenylephrine, the control treated with 0.1% DMSO. (*n* = 6; ^*∗∗*^*P* < 0.01, unpaired *t*-test).

**Figure 3:**
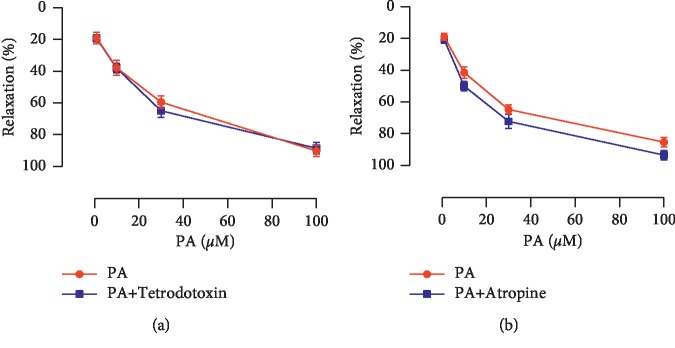
Relaxation effects of PA on endothelium-intact corpus cavernosum precontracted with phenylephrine in the absence and presence of (a) tetrodotoxin (1 *μ*M) and (b) atropine (30 *μ*M) (*n* = 10–11, *P* > 0.05 by paired *t*-test).

PA demonstrated a significant and concentration-dependent relaxation on rat corpus cavernosum strips precontracted by phenylephrine (Figure 2(a)). The maximal relaxation response to PA was 89.12% ± 4.62%, the EC_50_ was 20.48 *μ*M, and the curve was shown in Figure 2(b). After preincubation with tetrodotoxin to block the nerve conduction, similar magnitude relaxation was induced by PA (Figure 3(a)). Atropine also had no significant effect on the relaxation induced by PA (Figure 3(b)).

### 3.2. Role of Endothelium in the Relaxation Induced by PA

**Figure 4:**
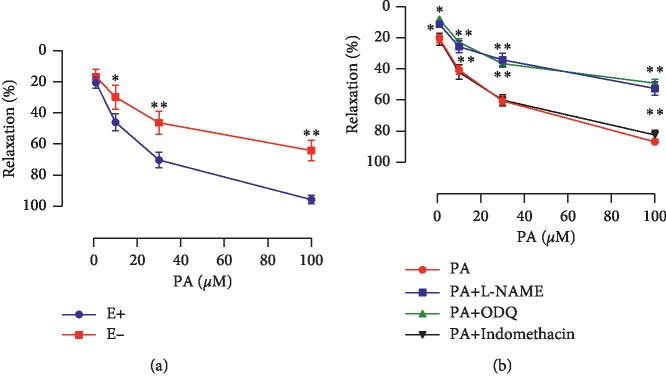
(a) Comparison of relaxation responses to PA in endothelium-intact (E+) and endothelium-deprived (E−) rat corpus cavernosum (*n* = 8, ^*∗*^*P* < 0.05, ^*∗∗*^*P* < 0.01, unpaired *t*-test). (b) Relaxation effects of PA on endothelium-intact corpus cavernosum precontracted with phenylephrine in the absence and presence of L-NAME (100 *μ*M), ODQ (10 *μ*M), or indomethacin (30 *μ*M) (*n* = 8–10; ^*∗*^*P* < 0.05 and ^*∗∗*^*P* < 0.01 compared with the PA group, paired *t*-test).

The PA-induced relaxation was reduced significantly after endothelium deprivation (Figure 4(a)), and the maximal relaxation decreased from 95.71% ± 1.16% to 64.04% ± 2.76% (*P* < 0.01). L-NAME and ODQ partially and significantly inhibited PA-induced relaxation compared with the PA group (*P* < 0.01), and the maximal relaxation decreased from 94.27% ± 3.97% to 54.46% ± 4.13% and to 49.15% ± 3.36% (Figure 4(b)), respectively. However, incubation with indomethacin had no effect on the PA-induced relaxation (Figure 4(b)).

### 3.3. Endothelium-Independent Relaxation Responses to PA

**Figure 5:**
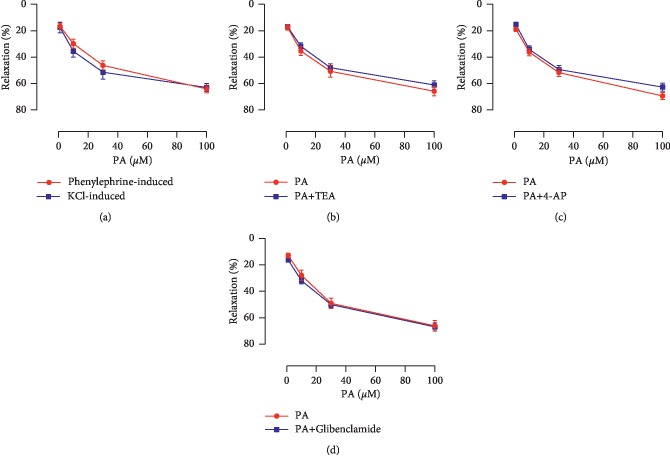
(a) Relaxation effects of PA against phenylephrine- and 60 mM KCl-evoked contraction in endothelium-deprived corpus cavernosum. Relaxation effects of PA on endothelium-deprived rat corpus cavernosum precontracted with phenylephrine in the absence and presence of (b) TEA (10 *μ*M), (c) 4-AP (300 *μ*M), or (d) glibenclamide (10 *μ*M) (*n* = 8–10, *P* > 0.05 by paired *t*-test).

PA demonstrated a significant and dose-dependent relaxation effect on corpus cavernosum precontracted by 60 mM KCl (Figure 5(a)).

After preincubation with Ca^2+^-activated K^+^ channels blocker TEA, voltage-dependent K^+^ channels blocker 4-AP, and ATP-sensitive K^+^ channels blocker glibenclamide, the maximal relaxation responses of endothelium-deprived corpus cavernosum to PA were 60.96% ± 2.95%, 62.64% ± 2.87%, and 62.71% ± 3.18%, respectively, which were similar to PA group (64.14% ± 3.67%) (Figures 5(b)–5(d)).

**Figure 6:**
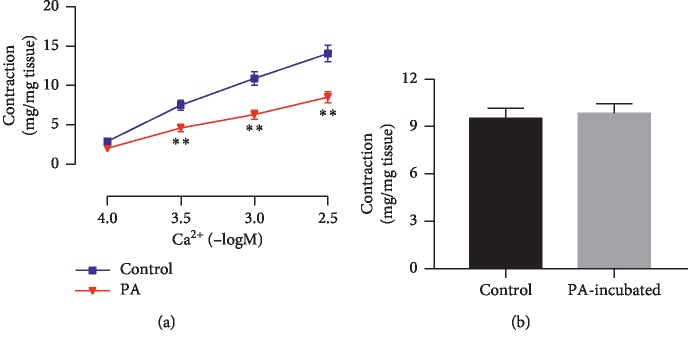
(a) Effect of PA (100 *μ*M) on endothelium-deprived corpus cavernosum contraction induced by cumulative addition CaCl_2_ in Ca^2+^-free high KCl (80 mM) solution. (b) Effect of PA (100 *μ*M) on endothelium-deprived corpus cavernosum contraction induced by phenylephrine in calcium-free solution (*n* = 8–10, ^*∗∗*^*P* < 0.01, paired *t*-test).

As shown in Figure 6(a), contractile response of endothelium-deprived corpus cavernosum induced by the cumulative addition of CaCl_2_ to Ca^2+^-free high potassium solution was significantly inhibited by PA (100 *μ*M) preincubation. *E*_max_ values of Ca^2+^-induced contractions with and without PA were 8.51 ± 0.68 and 14.06 ± 1.07 (mg/mg tissue) (*P* < 0.01). In Ca^2+^-free solution, phenylephrine-induced contraction in rat corpus cavernosum incubated with 100 *μ*M PA (9.52 ± 0.64 mg/mg tissue) was similar to vehicle control (9.84 ± 0.61 mg/mg tissue) (Figure 6(b)).

### 3.4. Effect of PA-Incubation on Sildenafil-Induced Relaxation

**Figure 7:**
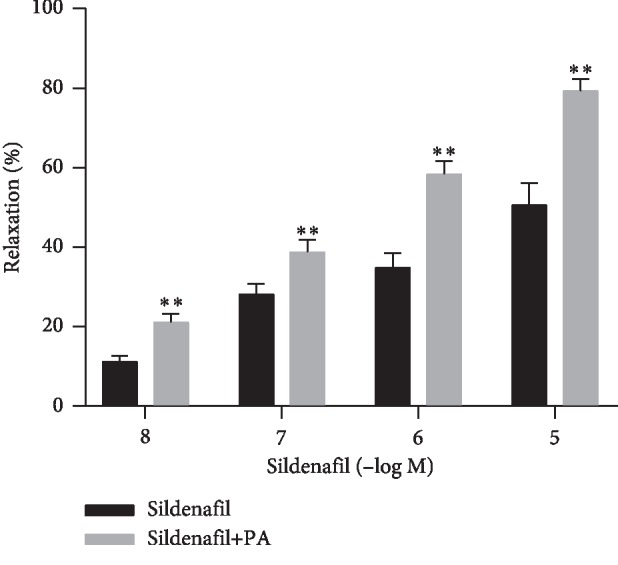
Effect of PA (20 *μ*M) incubation on rat corpus cavernosum concentration-dependent relaxation responses to sildenafil (0.01–10 *μ*M) (*n* = 8, ^*∗∗*^*P* < 0.01, paired *t*-test).

PDE-5 inhibitor sildenafil (0.01–10 *μ*M) demonstrated concentration-dependent relaxed rat corpus cavernosum. Relaxation response to sildenafil was significantly enhanced by PA (20 *μ*M) incubation (*P* < 0.01) (Figure 7).

### 3.5. Concentration of cGMP and cAMP

**Figure 8:**
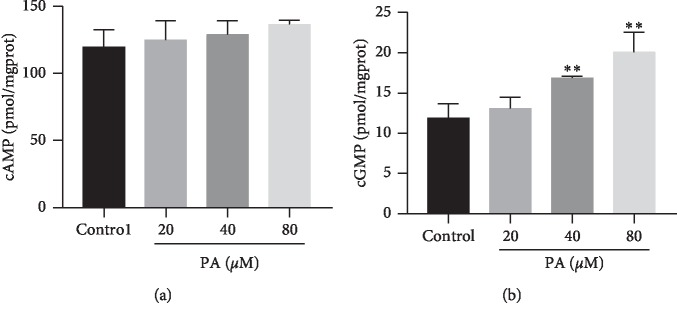
The levels of (a) cAMP and (b) cGMP in the rat corpus cavernosum after treatment with PA (20, 40, and 80 *μ*M) (*n* = 8 in each group, ^*∗∗*^*P* < 0.01 as compared with the control, one-way ANOVA).

Treatment with PA at 20, 40, and 80 *μ*M had no effect on the concentrations of cAMP in the corpus cavernosum tissues (Figure 8(a)). The concentrations of cGMP in the experimental groups treated with PA (40 *μ*M and 80 *μ*M) were 16.88 ± 0.21 and 20.05 ± 2.52 pmol·mg^−1^ (protein), which were significantly higher than that of the vehicle control group (11.87 ± 1.79 pmol·mg^−1^ (protein), *P* < 0.01). By contrast, treatment with 20 *μ*M PA had no effect on cGMP, 13.05 ± 1.43 pmol·mg^−1^ (protein) (Figure 8(b)).

### 3.6. Expression of nNOS and eNOS mRNA

**Figure 9:**
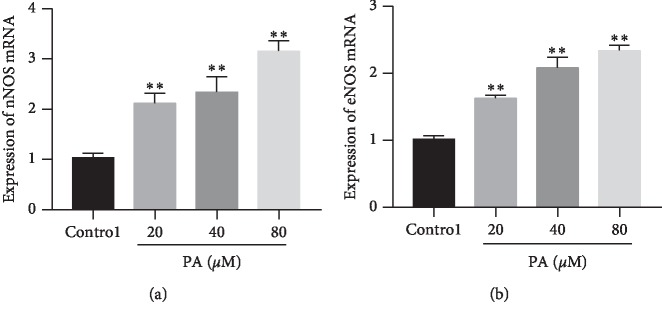
Expressions of nNOS (a) and eNOS (b) mRNA in rat corpus cavernosum after treatment with PA (20, 40, and 80 *μ*M) (*n* = 8; ^*∗∗*^*P* < 0.01 as compared with the control, one-way ANOVA).

Perfusion with PA (20, 40, and 80 *μ*M) significantly increased nNOS and eNOS mRNA expression (Figure 9).

### 3.7. Expressions of eNOS and nNOS

**Figure 10:**
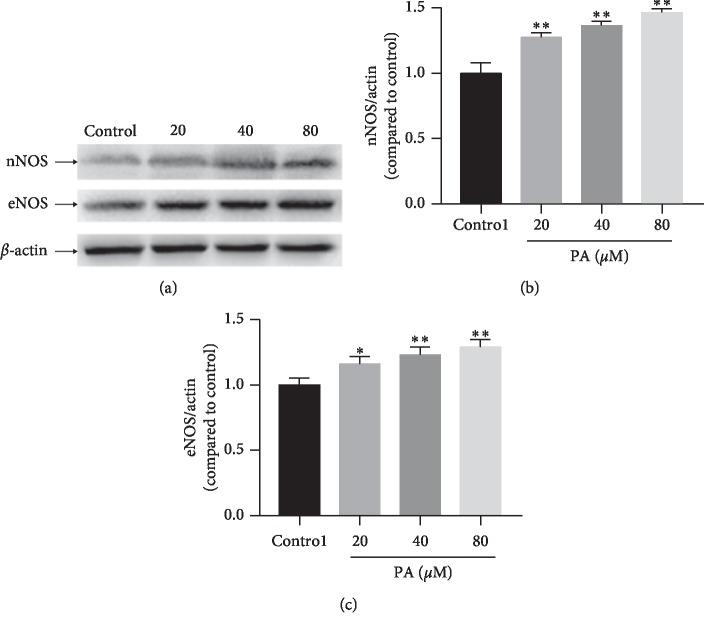
(a) Representative bands of protein. Expressions of nNOS (b) and eNOS (c) in rat corpus cavernosum after treatment with PA (20, 40, and 80 *μ*M) (*n* = 4; ^*∗*^*P* < 0.05, ^*∗∗*^*P* < 0.01 as compared with the control, one-way ANOVA).

Perfusion with PA (20, 40, or 80 *μ*M) significantly increased expression of nNOS and eNOS (Figure 10).

### 3.8. Effect of PA on ICP

**Figure 11:**
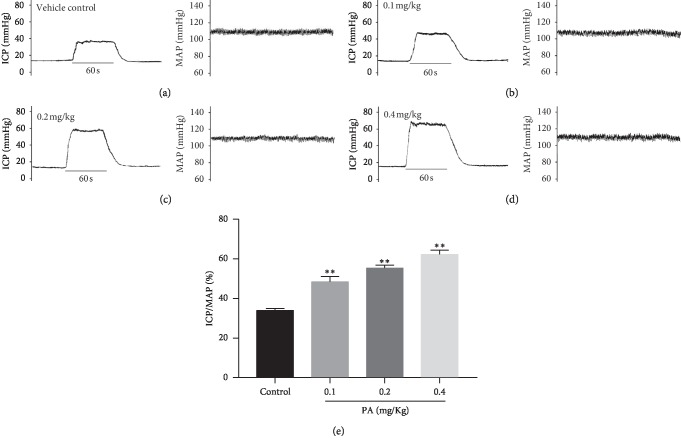
Original traces depicting MAP and ICP (a–d) of the control and PA groups (intracavernous injection with 0.1, 0.2, and 0.4 mg/kg PA) before and after electrical stimulation of the cavernous nerve. (e) Statistical chart of ICP/MAP ratio in each group during electrical stimulation (*n* = 6 in each group, ^*∗∗*^*P* < 0.01 as compared with the control, one-way ANOVA).

There were no significant differences in the MAP and basic ICP among the four experimental groups (data not shown). As shown in Figure 11, compared with the control group (33.91 ± 1.05), intracavernous injection of 0.1, 0.2, and 0.4 mg/kg PA significantly increased the ICP/MAP (%) (48.45 ± 2.62, 55.35 ± 3.18, and 62.25 ± 2.15, respectively) during the electrical stimulation of the cavernosum nerve.

## 4. Discussion

The present study, which was the first attempt to describe the corpus cavernosum relaxant effects of PA, demonstrated that PA had a significant concentration-dependent relaxant activity in the rat corpus cavernosum precontracted with phenylephrine. And the results suggested that PA induced relaxation of the corpus cavernosum strip by direct action on the corpus cavernosum, but not by a neuronal effect. Furthermore, muscarine was not involved. The relaxant effect of PA can be divided into endothelium-dependent and endothelium-independent mechanisms. The conclusion was based chiefly upon results showing that the relaxant effect of PA was significantly reduced by removing endothelium. The endothelium-dependent relaxant effect of PA seems to be attributable to the production of NO-like substances by the endothelial cells. The NO involved in penile erection is produced by NOS and is known to be an important biologically active substance involved in various physiological processes [20]. Released NO activates soluble guanylate cyclase to increase the production of cGMP, and the protein kinase cascade, hyperpolarization, and intracellular calcium sequestration promote the relaxation of the corpus cavernosum smooth muscle [21]. Finally, the arteries dilate, and blood influx increases. These results lead to increased penile pressure and penile erection [22]. In this study, PA-induced relaxation was significantly inhibited by the NOS inhibitor L-NAME and soluble guanylate cyclase inhibitor ODQ. The cyclooxygenase products, especially PGI_2_, as well as NO, play an important role in regulating the smooth muscle tone of the corpus cavernosum [23]. PGI_2_ may increase the concentration of cAMP in the corpus cavernosum smooth muscle cells by prostaglandin receptors stimulation [24, 25]. The binding of cAMP to the cAMP-dependent protein kinase PKA reduces the intracellular calcium levels [26] and leads to the relaxation of the corpus cavernosum [27]. However, in the present study, the cyclooxygenase inhibitor indomethacin had no effect on the PA-induced relaxation in endothelium-intact corpus cavernosum. Furthermore, PA failed to increase the level of cAMP in corpus cavernosum. These results indicated that the mechanism of endothelium-dependent was not involved in PGI_2_-pathway.

However, the partial relaxation effect of PA remained when the endothelium was removed or incubated with a NOS inhibitor. The results indicated that PA has a direct effect on the smooth muscle cells. The finding prompted us to investigate the response of endothelial-deprived corpus cavernosum to PA. The relaxant activity of PA in corpus cavernosum was not reduced in the high K^+^ medium. Given that the increase in tonic tension obtained in this experimental condition was due to extracellular Ca^2+^ influx after the opening of calcium channels [28], PA antagonized the Ca^2+^-induced contractions of the corpus cavernosum strips exposed to high KCl solution. This result suggested that the drug may be able to block the voltage-operated calcium channels, and it is consistent with a previous study in which PA had Ca^2+^ antagonist activity on Guinea pig colon [29]. K^+^ channels play an important role in regulating the corporeal smooth muscle cell tone, and the impairment of K^+^ channel activity may be one of the causes of erectile dysfunction [30]. However, several K^+^ channel blockers failed to affect the relaxation effect of PA on the corpus cavernosum smooth muscle, indicating that K^+^ channels were not involved in endothelium-independent mechanisms of action.

PDE-5 inhibitors, such as sildenafil, tadalafil, and vardenafil, are first-line drugs for the treatment of erectile dysfunction and have been evaluated as effective and safe [1, 31]. The total effective rate of these drugs was approximately 60%–70%, but in some patients, such as the sick suffering from severe neurological damage, radical prostatectomy, diabetes, or vascular disease, the total efficiency was markedly lower [32]. Therefore, finding new or alternative drugs for treating erectile dysfunction and the study of its mechanism of action remain significant. In our current experiments, PA increased the relaxant potency of PDE-5 on the corpus cavernosum and upregulated expression of mRNA and protein of nNOS and eNOS in the corpus cavernosum tissue. In addition, intracavernous injection of PA increased the ICP in a dose-dependent manner without significant effect on blood pressure. Therefore, the observed local penis effect of PA may be assumed to be not affected by systemic hemodynamic. Thus, the combination of less effective doses of sildenafil and PA has a potential advantage on erectile functions. Lower NOS mRNA expression was found in old rats, and NOS activity and content were reduced in rat models of both type I and II diabetes with ED [33]. We used healthy rats in this study before introducing a pathological condition such as diabetes mellitus or hypercholesterolemia; therefore further research using an animal model of ED is desirable.

In conclusion, in the present study, we demonstrated that PA has potent relaxation effects on the rat cavernosum smooth muscle through endothelium-dependent and -independent mechanisms. And intracavernous injection of PA increased the ICP/MAP. These results indicated that PA has the potential to be used as a drug for improving erectile dysfunction.

## Data Availability

The data used to support the findings of this study are available from the corresponding author upon request.

## Abbreviations

PA: patchouli alcohol
L-NAME: L-N^G^-nitroarginine methyl ester
ODQ: 1H-[1,2,4]oxadiazolo[4,3-a]quinoxalin-1-one
TEA: Tetraethylammonium
4-AP: 4-Aminopyridine
eNOS: Endothelial nitric oxide synthase
nNOS: Neuronal nitric oxide synthase
EGTA: Ethyleneglycol-bis-(b-amino-ethyl ether)-N,N,N′,N′-tetra-acetic acid
DMSO: Dimethyl sulphoxide
MAP: Mean arterial pressure
ICP: Intracavernous pressure.
